# Effects of eccentric exercises on improving ankle dorsiflexion in soccer players

**DOI:** 10.1186/s12891-021-04337-y

**Published:** 2021-05-26

**Authors:** Iris Femmigje Lagas, Duncan E. Meuffels, Edwin Visser, Floor P. Groot, Max Reijman, Jan A.N. Verhaar, Robert-Jan de Vos

**Affiliations:** 1 Department of Orthopaedic Surgery and Sports Medicine, Erasmus MC, University Medical Centre Rotterdam, Doctor Molewaterplein 40, 3015 GD Rotterdam, The Netherlands; 2 Department of Physiotherapy, Sportgeneeskunde Rotterdam, Rotterdam, The Netherlands; 3 Department of Sports Medicine, FIFA Medical Centre of Excellence, Royal Netherlands Football Association (KNVB), Zeist, The Netherlands

**Keywords:** Athletes, Exercise, Mass screening/methods, Wounds and injuries, Athletic injuries/prevention & control

## Abstract

**Purpose:**

The purpose of this study was to determine the effect of targeted eccentric calf muscle exercises compared to regular training on ankle dorsiflexion in healthy adolescent soccer players with a decreased ankle dorsiflexion.

**Methods:**

Male adolescent players (aged 14–21 years) from two professional soccer clubs were evaluated with the Weight Bearing Dorsiflexion Lunge Test (WBDLT) at baseline and after 12 weeks of this prospective controlled study. One club served as the control group and the other as the intervention group. Players with decreased ankle dorsiflexion (WBDLT) ≤ 10 cm) performed stretching and eccentric calf muscle exercises three times per week next to regular training in the intervention group, and performed only regular training in the control group. Primary outcome was the between-group difference in change in WBDLT between baseline and 12 weeks.

**Results:**

Of 107 eligible players, 47(44 %) had a decreased ankle dorsiflexion. The WBDLT (± standard deviation) increased in the intervention group from 7.1 (± 1.8) to 7.4 (± 2.4) cm (95 % Confidence Interval (CI)[-0.493 to 1.108], *p* = 0.381) and in the control group from 6.1 (± 2.4) to 8.2 (± 2.9) cm (95 % CI [1.313 to 2.659], *p* < 0.001). The difference in change of WBDLT between both groups was statistically significant (95 % CI [-2.742 to -0.510], *p* = 0.005).

**Conclusions:**

Targeted eccentric calf muscle exercises do not increase ankle dorsiflexion in healthy adolescent soccer players. Compared to regular training, eccentric exercises even resulted in a decreased calf muscle flexibility.

**Trial registration:**

This trial was registered retrospectively on the 7th of September 2016 in The Netherlands Trial Register (ID number: 6044).

## Background

Although Achilles tendinopathy is a persevering injury with low treatment response, research for prevention strategies are limited [1]. Achilles tendon injuries account for 2.4 % of all injuries in professional soccer players, and are associated with a prolonged absence in sports, work and other activities [2]. Besides the decrease in the athlete’s wellbeing and performance, the lack of a highly effective treatment for Achilles tendinopathy makes prevention essential [3].

Decreased ankle dorsiflexion increases strain on the soleus and gastrocnemius tendons. Gait analysis shows that soleus and gastrocnemius muscles absorb peak mechanical power just before toe-off in a walking and running gait cycle [4]. When ankle dorsiflexion is limited, the force absorbed by both soleus and gastrocnemius increases. Theoretically, this continuously increased strain can lead to Achilles tendinopathy. Two prospective studies reported that a decreased ankle dorsiflexion was associated with a 2.5–3.6 times higher risk of Achilles tendinopathy [5, 6].Consequently, ankle dorsiflexion angle is measured by many different medical professionals, such as physiotherapists, sports physicians and orthopaedic surgeons.

To reduce the risk of Achilles tendinopathy, stretching and eccentric (lengthening) exercises are postulated to improve ankle dorsiflexion. An eccentric exercise lengthens an active muscle while it is under load. By loading the Achilles tendon with eccentric (lengthening) exercises, lengthening of the musculotendinous junction may occur, leading to less strain on the tendon during movement [7]. Decreased plantar flexor muscle strength is also associated with a decreased ankle dorsiflexion [8]. Consequently, eccentric calf muscle exercises can also increase ankle dorsiflexion through an increase in calf muscle strength.

For the above mentioned reasons, a combination of stretching exercises and eccentric (lengthening) exercises are suggested as preventive intervention to increase ankle dorsiflexion. Our primary aim was to examine whether targeted stretching exercises and eccentric exercises of the calf muscles increase ankle dorsiflexion in healthy adolescent soccer players with a decreased ankle dorsiflexion. Our secondary aim was to determine the intra- and inter-observer reliability and minimal detectable change of the testing procedure.

## Methods

### Design

This prospective controlled trial was approved by the Medical Ethics Committee of the Erasmus MC Rotterdam, The Netherlands (MEC-2016-237). The trial is registered in the Netherlands Trial Register (NTR number: 6044). This study adheres to STROBE guidelines.

### Participants

Soccer players of Under-16, Under-17 and Under-19 squads of two Dutch professional premier division soccer clubs were asked to participate in this study. Before inclusion, informed consent was acquired from all players and parents (in case of players with age below 18 years). Players were eligible for inclusion if the following criteria were met: (1) age 14–21 years, (2) male sex, and (3) free of musculoskeletal injuries at baseline during physical testing. The soccer player was excluded if (1) he was not available in the week of baseline physical testing during the trial period.

Only players with a decreased ankle dorsiflexion were selected for further analysis. A decreased ankle dorsiflexion was defined as ≤ 10 cm toe-to-wall distance, or soleus muscle flexibility ≤ 34◦, or gastrocnemius muscle flexibility ≤ 34◦, measured with the WeightBearing Dorsiflexion Lunge Test (WBDLT) [6, 9]. Soccer players with a decreased ankle dorsiflexion from one club were assigned to the intervention group, and soccer players with a decreased ankle dorsiflexion from the other club were assigned to the control group. Both groups followed regular training, and the intervention group performed additional stretching and eccentric exercises for 12 weeks.

### Testing procedure and outcome measures

**Fig. 1:**
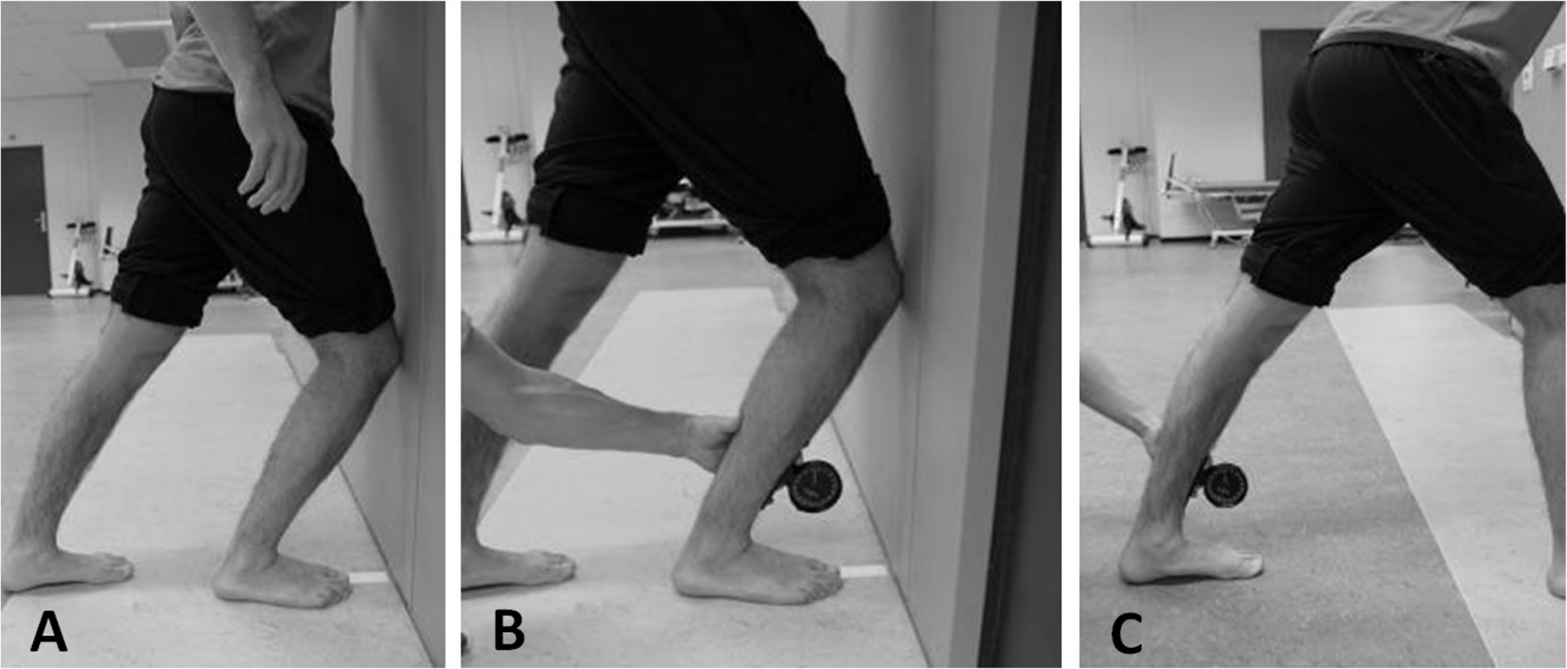
Outcome measures of ankle dorsiflexion. **a** Position of the weightbearing dorsiflexion lunge test. **b** Position for measuring the soleus muscle flexibility in degrees. **c **Position for measuring the gastrocnemius muscle flexibility in degrees

Age, body mass, height, and previous injuries were inquired with a baseline questionnaire. Testing procedures for calf muscle flexibility consisted of the WBDLT, the degree of soleus muscle flexibility and the degree of gastrocnemius muscle flexibility. The procedures for calf muscle flexibility were performed one week after the first training after the start of the soccer season for both teams. To ensure all players were fit, the tests were planned after a resting day. Before performing the WBDLT, a ruler was fixed on the floor (Fig. 1 a). A line was drawn on the shin 8 cm from the most prominent part of the distal fibula for placement of the lower part of the plurimeter (Dr. Rippstein, Zurich, Switzerland) The player stood barefooted with his heel and digit I of one foot aligned on the fixed ruler, facing the wall [6]. His knee was flexed with his patella against the wall above digit III. He was instructed to keep his trunk straight with his hands on his waist, and to dorsiflex his ankle as far as possible. We made sure the heel and digit I of the player were aligned with the fixed ruler. When the player could not further increase the ankle dorsiflexion, we noted down the distance between digit I and the wall. To measure soleus muscle length, a plurimeter was placed on the marked anterior tibial cortex, while the player stood in position A (Fig. 1b). The number of degrees between the tibial cortex and the floor was noted. To measure gastrocnemius muscle length, we instructed the player to stand in lunge position, while keeping his hind leg stretched (Fig. 1 c). The player bent his front leg to put strain on his fully extended knee joint. A plurimeter was placed on the marked anterior tibial cortex, while the player stood in position C. When the player could not further increase the ankle dorsiflexion of the hind leg, we noted the angle between the tibial cortex and the floor.

After every test, we asked the player if the limitation during dorsiflexion was felt on the anterior or dorsal side of the ankle (anterior blockage or dorsal tightness). The intervention group received their test results within one week after testing, with the aim to improve adherence to the intervention. The control group did not receive a report of their test results to prevent influencing their training habits as a consequence of their test results.

### Intervention

Both groups performed regular soccer training four times per week, with an approximate duration of 2 h per training. The intervention group was advised to perform targeted exercises after the soccer training. We instructed all players of the intervention group to perform the exercises three times a week for 12 weeks [10]. To ensure good performance of the exercises, they received detailed exercise instructions in groups by the principal investigator (IL) during the first week. Individual advice was given during the 12 weeks of exercising to ensure consistently good performance of the exercises.

**Fig. 2:**
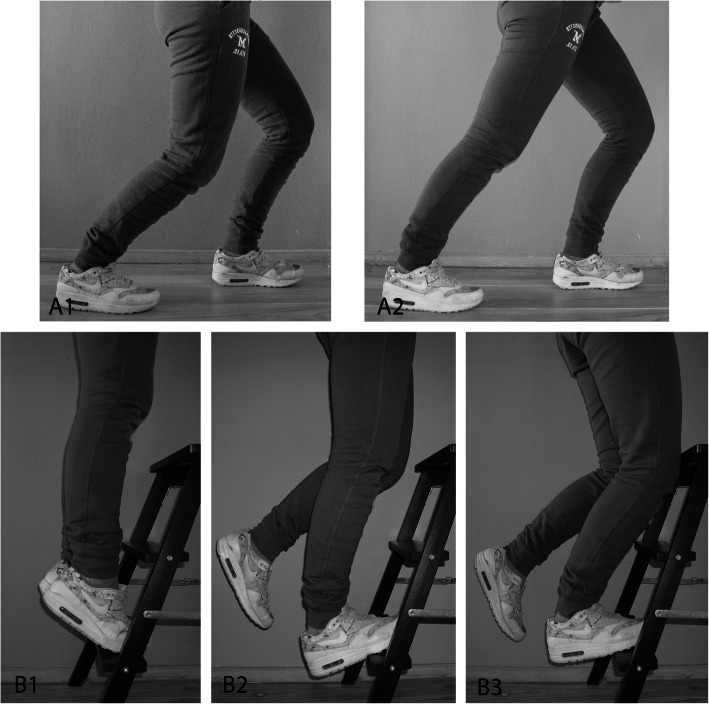
Stretching and eccentric (lengthening) exercises. A1) Stretching of the soleus muscle. A2 Stretching of the gastrocnemius muscle. B1) Starting position of eccentric exercises. B2) Eccentric exercise of the soleus muscle. B3) Eccentric exercise of the gastrocnemius muscles

Exercises aimed to lengthen the soleus and gastrocnemius muscles were selected (Fig. 2) [11]. For stretching of the soleus muscle, the player stood in a lunge position (Fig. 2A1). He then lowered his knee until he felt a stretch in the calf. For stretching of the gastrocnemius muscle, the player stood in a lunge position and flexed the knee of his front leg, while keeping both heels on the ground and the knee of his hind leg stretched (Fig. 2A2). Both stretching exercises (A1 and A2) were repeated three times for 30 s for the at-risk leg. The starting position of the eccentric exercises is showed in Fig. 2B1, where the balls of the players’ feet were on an elevation, with his heels above the ground. While keeping his posture straight, he slowly raised his heels until he was in a tiptoe position with both feet. For performing eccentric exercises of the soleus muscle, the player lifted up one leg from starting position so he stands on his to-be-trained leg (Fig. 2B2). He flexed his knee slightly and slowly lowered his heel until he feels a slight stretch. The other foot was placed on the elevation again and the exercise was repeated. For performing eccentric exercises of the gastrocnemius muscles, the player lowered his heel until he felt a slight stretch, while keeping his knee extended. The other foot was placed on the elevation again and the exercise was repeated.

The number of sets and repetitions was gradually increased by approximately 20 % every week to prevent overloading, starting at two times four repetitions (Fig. 2). If the ankle dorsiflexion was only decreased at one side, the players only performed the exercises for that index leg. If only the soleus muscle flexibility was limited, they only performed soleus lengthening exercises. One minute of rest between sets was advised. Primary outcome measure was the change in WBDLT after twelve weeks of training [12].

The sets for eccentric calf muscle exercises were repeated twice in the first four weeks, with four repetitions in the first week, increasing with two repetitions per week. In week five to eleven, sets were repeated three times, starting with seven repetitions, and increasing with one repetition per week. Three sets of 15 repeats were accomplished in week twelve.

One researcher (IL) registered individual compliance of the soccer players to the exercises by attending every training and observing the performance of the exercises. The compliance was calculated by dividing the performed number of exercises to the number of prescribed exercises, represented as percentages.

One researcher (IL) visited the physiotherapists of both clubs weekly for registration of Achilles tendon injuries. An Achilles tendinopathy was defined as a focal physical complaint of the Achilles tendon with pain on palpation leading to the athlete being unable to take part in training and/or competition [13].

### Statistical analysis

SPSS software (V.21.0; SPSS, Chicago, Illinois, USA) was used for statistical analysis. In case of missing data at the 12 weeks’ time point, the data was described as ‘missing’ in the analysis. The within-group changes in WBDLT, soleus and gastrocnemius muscle flexibility were analysed with a paired sample T-test and described as mean ± standard deviation. The change in centimetres on the WBDLT and degrees on the soleus and gastrocnemius muscle flexibility tests were analysed with a linear regression analysis. In an univariate model, we analysed if there was an association between baseline characteristics and the change in ankle dorsiflexion. If one of these variables had an association with a p-value < 0.10, this variable was included in a multivariate stepwise regression. To calculate the correlation between the change in centimetres on the WBDLT outcome and degrees on the soleus and gastrocnemius muscle flexibility tests and compliance to the targeted training programme, a Pearson correlation test was used. A p-value < 0.05 was considered as statistically significant in the final analyses.

To ensure the reliability of the testing procedures for calf muscle flexibility, intra-class correlation coefficient (ICC) for the intra- and inter- observer reliability and minimal detectable change (MDC) were examined. The WBDLT was performed by ten healthy male participants. The test was independently instructed and measured by two researchers (RJdV, FG). One of these researchers (FG) performed the same test one day later. ICC values for intra- and inter-observer reliability were interpreted according to Fleiss as poor (< 0.40), fair (0.40–0.59), good (0.60–0.75) and excellent (> 0.75) [14]. MDC was calculated with the formula $MDC=SEM*1.96* \surd 2$, where the standard error of the mean (SEM) was $SD*\sqrt{1-ICC}$. The standard deviation (SD) was the SD of all scores from the participants [15].

## Results

### Baseline player characteristics

**Fig. 3:**
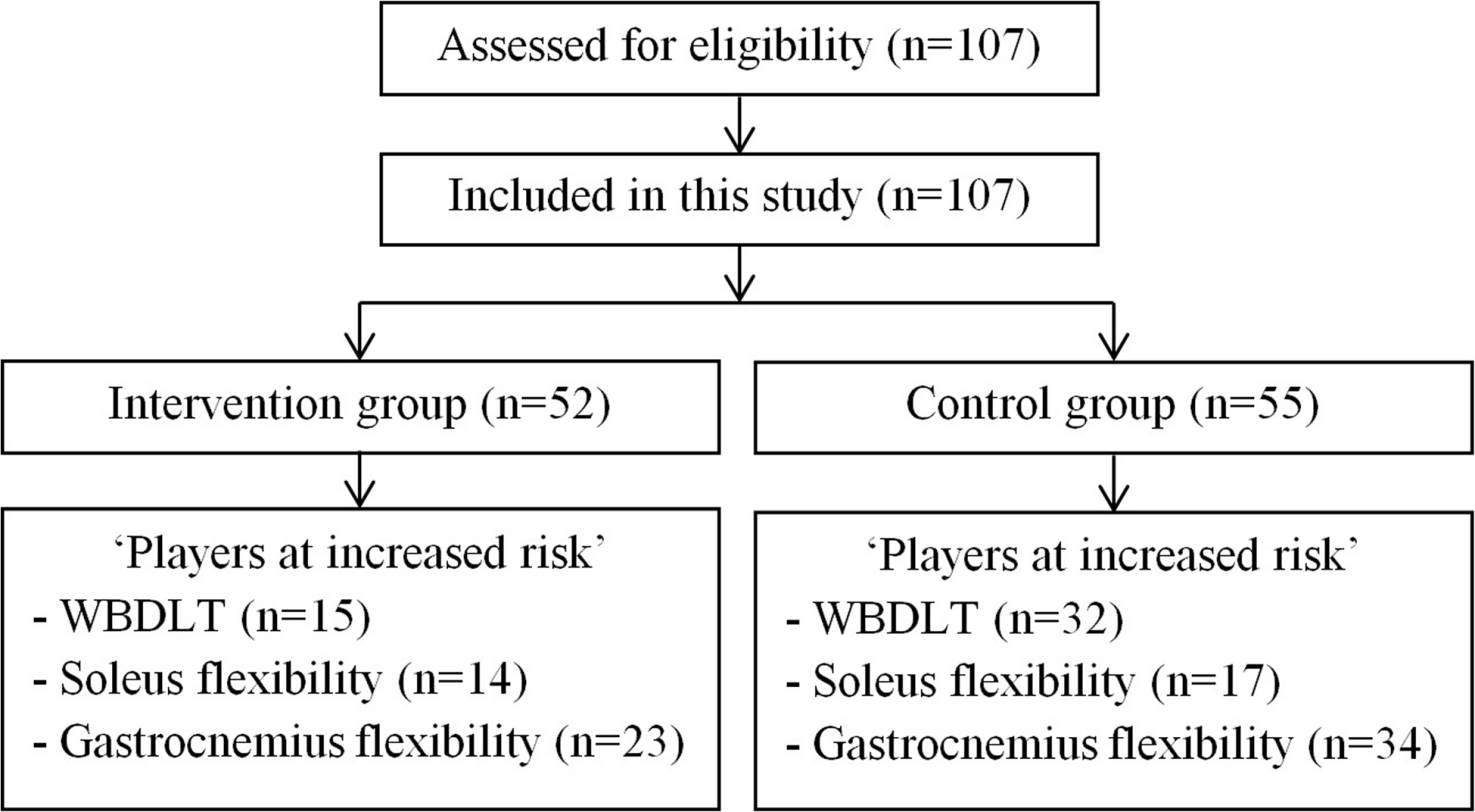
Flowchart of patients through the study

In total, 107 players were assessed for eligibility in July and August 2016, and could be included in this study (Fig. 3). There was no exclusion of players. Two weeks after baseline testing, the intervention group started the training programme. Baseline characteristics of players with a decreased ankle dorsiflexion (WBDLT of ≤ 10 cm, soleus muscle flexibility ≤ 34◦, or gastrocnemius muscle flexibility ≤ 34◦) are presented in Table 1. No Achilles tendon injuries occurred in both groups during the exercise period and there was no loss to follow-up.

**Table 1:** Baseline statistics of players at increased risk of Achilles tendinopathy

	Intervention group	Control group	*p*-value
Age, years	16.3 ± 1.2	16.3 ± 1.2	0.997
Height, meters	1.76 ± 0.07	1.76 ± 0.09	0.992
Weight, kg	65.6 ± 8.1	68.0 ± 10.5	0.344
BMI, kg/m^2^	21.1 ± 1.8	21.7 ± 2.2	0.207
Weekly training, hours	9.4 ± 2.4	9.0 ± 2.2	0.490

Data is presented as mean ± SD

* Statistically significant difference (*p*-value < 0.05). *SD* Standard deviation, *BMI *Body Mass Index

### Weightbearing dorsiflexion lunge test

The mean WBDLT in the intervention group improved from 7.1 (± 1.8) to 7.4 (± 2.4) cm (*p* = 0.381). In the control group mean WBDLT improved from 6.1 (± 2.1) to 8.2 (± 2.9) cm (*p* < 0.001). The difference in change of WBDLT between both groups was statistically significant (95 % CI [-2.7 to-0.5], *p* = 0.005). Neither baseline characteristics, presence of anterior blockage or dorsal tightness or baseline influenced the magnitude of change in WBDLT. There was no significant association between the intervention and change in WBDLT (Table 2).

**Table 2:** Multivariate linear regression analysis of change in WBDLT after 12 weeks

Test	Variables	Unstandardized Beta [95 %CI]	p-value
WBDLT^a^	Intervention	1.626 [0.510;2.742]	0.005*
Soleus muscle flexibility^b^	Intervention	3.598 [0.992;6.205]	0.009*
	Age (years)	-0.987 [-2.156;0.183]	0.095
	BMI (kg/m^2^)	-0.150 [-0.748;0.447]	0.609
	Anterior limitation	0.000 [0.000;0.001]	0.158
Gastrocnemius muscle flexibility^c^	Intervention	1.578 [-0.997;4.132]	0.221
	Anterior blockage	0.852 [-2.389;4.094]	0.600

^a^*r*^2^ = 0.171

^b^*r*^2^ = 0.338

^c^*r*^2^ = 0.029

*Statistically significant difference (*p*-value < 0.05)

*CI* Confidence Interval

### Soleus and gastrocnemius muscle flexibility

In the intervention group, mean soleus muscle flexibility improved from 31.0 (± 1.7) to 32.5 (± 3.3) degrees (*p* = 0.075). The mean soleus muscle flexibility of the control group had a statistically significant improvement from 28.3 (± 3.4) to 33.6 (± 4.7) degrees (*p* < 0.001). The baseline value differed between both groups (95 % CI [0.7 to 4.7], *p* = 0.011).

Age and BMI were univariably associated with a positive influence on change in soleus muscle flexibility. Older players and players with a higher BMI had a larger improvement in soleus muscle flexibility (Table 2). However, neither age (*p* = 0.095) and BMI (0.609) were predictors of change in soleus muscle flexibility in a multivariable analysis. Other baseline characteristics, presence of anterior blockage or dorsal tightness did not influence the magnitude of change in soleus muscle flexibility. The intervention was significantly associated with a decrease in soleus muscle flexibility (*p* = 0.009) (Table 2).

Mean gastrocnemius muscle flexibility of the intervention group improved from 29.8 (± 3.0) to 31.0 (± 3.5) degrees (*p* = 0.188).The mean gastrocnemius muscle flexibility of the control group improved significantly from 28.3 (± 4.4) to 31.2 (± 5.6) degrees (*p* = 0.004). There was no significant relation between the intervention and the change in gastrocnemius muscle flexibility (Table 2). Neither baseline characteristics, presence of anterior blockage or dorsal tightness influenced the magnitude of change in gastrocnemius muscle flexibility.

### Compliance to exercises

The mean compliance to the exercise program was 69 (± 14) % for WBDLT, 67.4 (± 14.6) % for soleus muscle flexibility, and 63.9 (± 16.4) % for gastrocnemius muscle flexibility. This means that on average, all players performed the exercises twice per week. The compliance of individual players did not significantly correlate with their corresponding change in WBDLT result (*r*=-0.313, *p* = 0.275) and soleus muscle flexibility(*r*=-0.411, *p* = 0.163). Compliance of individual players to stretching and eccentric exercises were correlated with their corresponding change in gastrocnemius muscle flexibility (*r* = 0.474, *p* = 0.022).

### Reliability of testing procedure

The intra-observer reliability of the WBDLT was 0.98 (95 % CI [0.94 to 0.99]), 0.95 (95 % CI [0.76 to 0.96]) for soleus muscle flexibility and 0.94 (95 % CI [0.86 to 0.98]) for gastrocnemius muscle flexibility. The inter-observer reliability was determined to be 0.99 (95 % CI [0.999 to 0.999]) for the WBDLT, 0.98 (95 % CI [0.94 to 0.99]) for soleus muscle flexibility and 0.98 (95 % CI [0.94 to 0.99]) for gastrocnemius muscle flexibility. All can be defined as excellent agreement. The MDC for WBDLT was 1.5 cm, 4.7 degrees for soleus muscle flexibility and 4.9 degrees for gastrocnemius muscle flexibility.

## Discussion

Our study is the first to compare the effect of stretching and eccentric (lengthening) exercises with regular training on ankle dorsiflexion in a population of adolescent soccer players a decreased ankle dorsiflexion. Contrary to popular belief, ankle dorsiflexion did not improve after targeted stretching and eccentric exercises of the calf muscles.

Only one preventive intervention study has been performed in this field. A large Danish study showed that eccentric exercises as prevention had no influence on ultrasonographic abnormalities of the Achilles tendon [16]. In addition, stretching and eccentric exercises did not reduce the risk of developing symptoms of Achilles tendinopathy [16]. Our study provides a possible explanation for these results; stretching and eccentric exercises do not increase the limited ankle dorsiflexion and thereby do not influence a potential risk factor.

As a decreased ankle dorsiflexion is associated with a higher risk of Achilles tendinopathy [6], we used exercises that were thought to increase ankle dorsiflexion. As no prevention strategy is yet developed for Achilles tendon injuries, we looked at possible ways to increase the ankle dorsiflexion. Tendon length is often associated with the concept of tendon stiffness [17]. It is hypothesized that it is desirable to make a muscle tendon unite more flexible (resulting in a larger ankle dorsiflexion angle). However, results vary in literature. In one study, patients with Achilles tendinopathy were included and randomized to either a 12-week eccentric calf muscle program or a control group [18]. There was no significant increase in dorsiflexion range of motion in the eccentric loading group. In another study by Mahieu et al., the ankle dorsiflexion angle increased after a 6-week eccentric training regime in healthy subjects when compared to the control group [19]. The differences between these studies might be explained by differences in included population; one included patients and the other healthy subjects [18, 19]. As we also included healthy subjects, it is striking that our study results were opposite to the study results of Mahieu et al. [19] A reason for this might be the duration of the intervention. A recent systematic review showed that stretching exercising protocols shorter than 8 weeks do not change either muscle or tendon properties [20]. It is hypothesized that eccentric exercises result in changes at a sensory level in the short term and that tissue properties change on the long term.

From a mechanistic perspective, we hypothesized that hypothesized stretching and eccentric exercises would increase calf muscle flexibility through (1) induction of sarcomerogenenesis [21], (2)lengthening of the myotendinous unit [7, 22] and (3) strengthening of the plantar flexors [8]. However, we did not find a correlation between compliance to exercises and change in ankle dorsiflexion. It is, based on our study results, unknown whether this is caused by decreased muscle flexibility, increased Achilles tendon stiffness or a combination of both. A recent study shows that an 8-week eccentric exercise program stimulates increased cross-sectional area of the tendon (hypertrophy) and simultaneous increased stiffness in healthy subjects [23]. The increased stiffness might be explained by a loss of collagen crimp or increased crosslinking of the tendon fibrils [24, 25]. These mechanistic effects might explain our study findings.

We also investigated whether the effect of the intervention could be explained by baseline parameters. If a player is limited in ankle dorsiflexion due to blockage at the anterior side of the ankle, it is less likely that stretching and eccentric calf muscle exercises can influence the ankle dorsiflexion. However, both WBDLT and soleus muscle flexibility were not influenced by anterior blockage of ankle dorsiflexion. Feeling of calf muscle tightness during testing of the gastrocnemius muscle flexibility was associated with improvement of gastrocnemius muscle flexibility in the univariate regression analysis, but showed no statistically significant association in the multivariate regression analysis. Other baseline variables were also not associated with an improved ankle dorsiflexion. We did not find confounders that might have altered the effectiveness of the exercises.

The strengths of our study are the implementation of an adequate methodology and the fact that we adhered to the predefined study protocol. Before the testing moments, a clear consensus was made between all researchers. This ensured that during the testing moments, the same instructions were given to all soccer players with the aim to improve reliability of the testing procedure. This was reflected by the excellent intra-observer and inter-observer reliability of the performed tests. MDC values were low, which means that we were able to demonstrate clinically relevant changes outside the measurement error. This was true for the WBDLT, as the difference in change between both groups was outside the measurement error. However, the difference in change for soleus and gastrocnemius muscle flexibility was within the MDC and therefore the clinical relevance of the findings related to the soleus and gastrocnemius muscle flexibility is limited.

There are some limitations to our study. First, we determined clusters by the club at which a player was training, to avoid contamination of the intervention. However, because we used different clubs, it could be possible that the two clusters had different training regimes with a different total exercise time, although training frequency was equal. Both clubs followed our regulations to not alter their regular training, and not to perform extra prevention exercises. Our results showed that the player characteristics were not significantly different between both clusters, meaning that they were comparable. The baseline WBDLT and gastrocnemius muscle flexibility was similar in both groups, but the soleus muscle flexibility showed a significant difference at baseline; the control group had smaller soleus muscle flexibility than the intervention group. However, this difference was within the measurement error. We chose to use cluster randomisation for practical reasons. Nevertheless, there are systematic biases associated with cluster randomisation. The presence of selection bias can occur with cluster designs, such as age imbalance between clusters. While we cannot exclude occurrence of bias in our study, the between-group differences in baseline characteristics were similar.

Another limitation could be the cut-off of all tests. Although we based our cut-offs on previous studies [6, 9], we are aware that the cut-offs are not determined by a large prospective trial. Unfortunately, both studies did not publish sensibility and sensitivity of the cut-offs, which adds to the limitation. A cut-off value for WBDLT and soleus muscle flexibility was needed to determine which group had a decreased dorsiflexion. We chose this approach, because we expected a better effect of the eccentric exercises in this subgroup.

Last limitation could be the moderate compliance to the exercises. In order for a prevention strategy to be effective, exercises should be performed at least twice a week [11]. We made our intervention group perform the exercises three times a week, thus making sure they performed enough exercises in order for the intervention to be effective. The average compliance to exercises is approximately 66 % of prescribed exercises (thus three times a week). This equals to an average performance of two times a week per player. We should be aware that the moderate compliance is at least a reflection of daily clinical practice.

### Practical Applications

Stretching and eccentric (lengthening) exercises are generally advised as they are thought to improve the ankle dorsiflexion, a risk factor for developing Achilles tendon injuries [6]. However, the findings of this study demonstrate that stretching and eccentric (lengthening) exercises do not increase ankle dorsiflexion in adolescent high level soccer players with a decreased ankle dorsiflexion compared to regular training. The outcome of this study questions whether stretching and eccentric (lengthening) exercises should be used as prevention exercises. Future studies should be aimed at novel methods to improve ankle dorsiflexion.

## Conclusions

Stretching and eccentric exercises do not increase ankle dorsiflexion in adolescent high level soccer players. Compared to regular training, eccentric exercises even resulted in a decreased calf muscle flexibility. This might explain why targeted eccentric calf muscle exercises are not effective as primary preventive intervention for Achilles tendon injuries.

## Data Availability

The datasets used and analysed during the current study are available from the corresponding author on reasonable request.
